# Perioperatives Flüssigkeitsmanagement bei großen viszeralchirurgischen Eingriffen

**DOI:** 10.1007/s00101-020-00867-7

**Published:** 2020-10-09

**Authors:** M. von der Forst, S. Weiterer, M. Dietrich, M. Loos, C. Lichtenstern, M. A. Weigand, B. H. Siegler

**Affiliations:** 1 Klinik für Anästhesiologie, Universitätsklinikum Heidelberg, Im Neuenheimer Feld 110, 69120 Heidelberg, Deutschland; 2 Klinik für Anästhesie und operative Intensivmedizin, Rheinland Klinikum Neuss/Lukaskrankenhaus, Preußenstraße 84, 41464 Neuss, Deutschland; 3 Klinik für Allgemein‑, Viszeral- und Transplantationschirurgie, Universitätsklinikum Heidelberg, Im Neuenheimer Feld 110, 69120 Heidelberg, Deutschland

**Keywords:** Flüssigkeitstherapie, Viszeralchirurgie, Perioperatives Management, Euvolämie, Hämodynamische Überwachung, Fluid therapy, Visceral surgery, Perioperative management, Euvolemia, Hemodynamic monitoring

## Abstract

Die Gabe intravasaler Flüssigkeiten gehört zu den Grundpfeilern der perioperativen Therapie und nimmt insbesondere bei großen viszeralchirurgischen Eingriffen maßgeblichen Einfluss auf das chirurgische Behandlungsergebnis. Ein adäquates perioperatives Flüssigkeitsmanagement kann durch Vermeidung von Hypo- und Hypervolämie dazu beitragen, das Risiko einer unzureichenden Gewebeperfusion als Treiber postoperativer Morbidität und Letalität signifikant zu reduzieren. Der effektive Umgang mit intravasal zugeführten Flüssigkeiten setzt dabei die Kenntnis der Substanzen sowie Maßnahmen zur Therapiesteuerung voraus. Das Flüssigkeitsmanagement beginnt bereits präoperativ und sollte – unter Nutzung einer an die Bedürfnisse des Patienten angepassten und dem Eingriff entsprechenden hämodynamischen Überwachung – auch postoperativ im Aufwachraum und auf Station fortgesetzt werden. Der Kommunikation aller an der perioperativen Versorgung Beteiligten kommt im Sinne eines optimalen Flüssigkeitsmanagements eine entscheidende Bedeutung zu.

## Abstract (en)

Intravascular fluid administration belongs to the cornerstones of perioperative treatment with a substantial impact on surgical outcome especially with respect to major abdominal surgery. By avoidance of hypovolemia and hypervolemia, adequate perioperative fluid management significantly contributes to the reduction of insufficient tissue perfusion as a determinant of postoperative morbidity and mortality. The effective use of intravascular fluids requires detailed knowledge of the substances as well as measures to guide fluid therapy. Fluid management already starts preoperatively and should be continued in the postoperative setting (recovery room, peripheral ward) considering a patient-adjusted and surgery-adjusted hemodynamic monitoring. Communication between all team members participating in perioperative care is essential to optimize fluid management.

Man kann die Erkenntnisse der Medizin auf eine knappe Formel bringen: Wasser, mäßig genossen, ist unschädlich. Mark Twain [7]

Die Gabe intravasaler Flüssigkeiten ist aus heutiger Sicht ein kaum mehr wegzudenkendes Element des medizinischen Alltags. Bereits vor der ersten Äthernarkose wurden im Rahmen einer Choleraepidemie Therapieversuche mit i.v.-Flüssigkeitsgabe unternommen; eine Maßnahme, die sich aufgrund des klinischen Erfolgs rasch auch im Kontext chirurgischer Eingriffe etablierte [79]. Die Möglichkeit des Ausgleichs intraoperativer Blutverluste bildete zusammen mit der (Weiter‑)Entwicklung von Infusionslösungen – später ergänzt durch die Transfusionsmedizin – die Basis für immer komplexere chirurgische Prozeduren an zunehmend multimorbiden Patientenkollektiven.

Wenngleich der Kliniker diesen Herausforderungen heute mit vielfältigen Möglichkeiten zu Aufrechterhaltung und Überwachung der Herz-Kreislauf-Funktion begegnen kann, bleibt der perioperative Einsatz von Flüssigkeiten nach wie vor Gegenstand zahlreicher Untersuchungen und wissenschaftlicher Diskussionen. So werden die Begriffe „Flüssigkeitstherapie“ und „zielgerichtete Therapie“ („goal-directed therapy“ [GDT]) in einer Analyse zum Forschungsbedarf in der perioperativen Phase an erster und dritter Stelle der 10 wichtigsten Forschungsthemen für die nächsten 10 Jahre aufgeführt [32]. Die Relevanz dieser Themen wird durch Ergebnisse aktueller Untersuchungen deutlich, wonach sich die Wahl des perioperativen Flüssigkeitsregimes über kurz- und mittelfristige Folgen hinaus auch auf die Langzeitletalität auszuwirken scheint [8].

Auswertungen der anwenderspezifischen Flüssigkeitsgabe bei viszeralchirurgischen Eingriffen zeigen eine enorme Streubreite zwischen einzelnen Anästhesisten [71]. Zwar existieren Leitlinien zur intravasalen Flüssigkeitstherapie sowohl im englischsprachigen als auch deutschen Raum, diese können spezielle Patientenkollektive jedoch nur bedingt berücksichtigen [83, 96]. Entsprechend der Datenlage hält zunehmend die Erkenntnis Einzug, dass sich eine möglichst individualisierte Therapie am ehesten als zielführend erweist. Im klinischen Alltag bietet sich in diesem Kontext wohl eine Unterteilung nach Patientenrisiko und der Art des Eingriffs an, um eine bestmögliche Kombination von Individualisierung und Standardisierung zur erreichen [92, 97].

Die vorliegende Arbeit fokussiert auf das Patientenkollektiv der großen elektiven Viszeralchirurgie. Sowohl Transplantationen als auch akute Krankheitsbilder (Sepsis, akute Pankreatitis) werden in diesem Rahmen nicht behandelt, da hier ein gesondertes Flüssigkeitsmanagement erforderlich ist.

## Grundlagen

**Figure 1:**
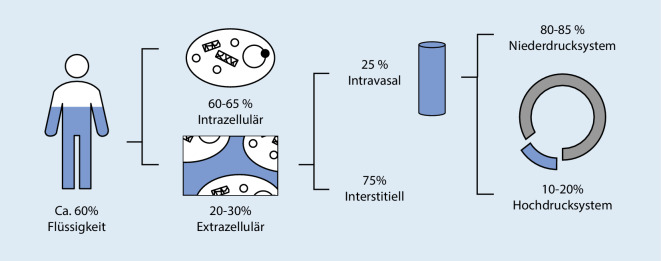

Der menschliche Organismus besteht, abhängig von Alter und Gesundheitszustand, zu etwa zwei Dritteln aus Wasser. Grundsätzlich wird zwischen intra- und extrazellulärer Flüssigkeit unterschieden. Letztere verteilt sich zu einem Großteil im interstitiellen Raum; lediglich eine kleine Menge befindet sich intravasal (Abb. 1).

Unter alltäglichen Bedingungen muss der Organismus v. a. Verluste durch Atmung (Perspiratio), Schwitzen, Fäzes und Urinausscheidung kompensieren, diese werden regelhaft über die orale Flüssigkeitszufuhr ausgeglichen. Sofern dies nicht ausreicht, greift der Körper auf verschiedene Regelkreise zurück. Kurzfristig erfolgt eine Regulation über die Herzfunktion und den Gefäßmuskeltonus und langfristig über den Wasser-Elektrolyt-Haushalt. Bedeutender Akteur in diesem Kontext ist das Renin-Angiotensin-Aldosteron-System. Angiotensin II fördert die Aldosteron- sowie die Vasopressinausschüttung und wirkt u. a. auch durch direkte Vasokonstriktion. Sowohl Vasopressin als auch Aldosteron beeinflussen den Elektrolythaushalt, insbesondere die Natriumkonzentration, welche entscheidend zur Osmolarität des Blutes und damit ebenso wie körpereigene Proteine, z. B. Albumin, zur Flüssigkeitsverteilung beiträgt. Vasopressin wirkt über die Aktivierung der V_2_-Rezeptoren und den konsekutiven Einbau von Aquaporin 2 mit Resorption von „freiem“ Wasser in der Niere. Darüber hinaus erhöht es auch den Gefäßmuskeltonus [65, 120], wohingegen Aldosteron über den Mineralokortikoidrezeptor die Natriumrückresorption im Tubulussystem fördert. Im perioperativen Kontext erfolgt eine Beeinflussung insbesondere der kurzfristigen Regelkreise, z. B. mittels Katecholaminen oder Vasopressinanaloga. Ein neuer Ansatz ist z. B. der Einsatz von Angiotensin II zur Blutdrucksteuerung im vasodilatativen Schock [59].

## Perioperative Einflussfaktoren

**Figure 2:**
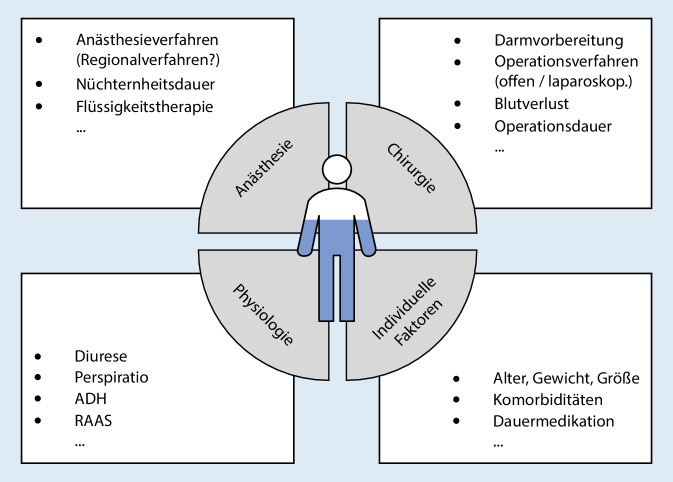

Ein optimales perioperatives Flüssigkeitsregime setzt die Berücksichtigung individueller Faktoren und der chirurgischen Intervention voraus (Abb. 2). Zu individuellen Faktoren zählen Alter und Ernährungszustand, aber auch erkrankungsbedingte Flüssigkeitsverschiebungen, welche sich beispielsweise als Ödeme oder Aszites bei Herz- bzw. Leberinsuffizienz äußern. Weiterhin muss bei terminalem Nierenversagen, schlecht eingestelltem Diabetes mellitus, Diarrhö oder Erbrechen von einem nichtausgeglichenen Flüssigkeitshaushalt ausgegangen werden.

Darüber hinaus greifen verschiedene, dem Eingriff geschuldete Maßnahmen in den Flüssigkeitshaushalt ein. Aktuelle Leitlinien der *American Society of Anesthesiologists *(ASA) empfehlen einen Nahrungsverzicht ab 6 h präoperativ und eine komplette Nüchternheit ab 2 h vor der Operation [5]. Einerseits konnte gezeigt werden, dass die Nüchternheitsphase nicht zu einem Flüssigkeitsdefizit führt [39], andererseits ist zu berücksichtigen, dass es organisations- oder patientenbedingt im Alltag potenziell zu einer Überschreitung der empfohlenen Intervalle (z. B. bei verschobenen Elektiveingriffen oder weil der Operationszeitpunkt nicht absehbar ist) kommen kann. Zwar konnten Jacob et al. bei kardiopulmonal gesunden Patienten auch nach über 10 h Fasten keinen Flüssigkeitsmangel nachweisen, ob dies auf die zunehmend multimorbiden Patienten übertragbar ist, bleibt hingegen fraglich [49]. Eine präoperative Darmvorbereitung mittels Laxanzien mit den entsprechenden Konsequenzen für den Flüssigkeitshaushalt wird aufgrund der unerwünschten Nebeneffekte v. a. in der *Fast-Track*-Chirurgie nicht mehr routinemäßig empfohlen [131].

Auch die Wahl des Narkoseverfahrens muss bei der Flüssigkeitskalkulation berücksichtigt werden. Sowohl Vollnarkosen als auch rückenmarknahe Verfahren können mit einer u. a. durch Sympathikolyse verursachten Vasodilatation und entsprechender Flüssigkeitsverschiebung einhergehen [42]. Zusätzlich verstärkt die Kombination von Peridural- und Allgemeinanästhesie diesen Effekt und kann zu einem erhöhten Volumenbedarf führen [146].

Intraoperativ sind wie beim wachen Patienten physiologische Flüssigkeitsverluste durch Urinausscheidung und Perspiratio zu erwarten. In den britischen Leitlinien und verschiedenen Studien werden zur reinen Erhaltungstherapie etwa 1,0–3,0 ml/kgKG und h empfohlen; hinzukommen können außerdem Verluste durch extremes Schwitzen oder über die Drainagen abgeleitete Sekrete [78, 94, 96].

Durch chirurgische Intervention verursachte Flüssigkeitsverschiebungen, bei Manipulation des Darms oder des Mesenteriums, das Leeren von Zysten oder großer Mengen Aszites sowie Blutverluste führen ebenfalls zu teilweise erheblichen Veränderungen des Flüssigkeitshaushaltes. In diesen Kontext fallen auch temporäre Gefäßverschlüsse (bei Resektion oder zur Blutungskontrolle), welche mit einer konsekutiven Einschwemmung von Stoffwechselprodukten und einer relevanten Flüssigkeitsverschiebung einhergehen können. Insbesondere müssen auch Verluste durch Exenteration von viszeralen Organen ersetzt werden, welche mit Werten von 5,46 bis 19,6 ml/kgKG und h bei normalgewichtigen Patienten (70 kg, 170 cm) deutlich variieren [66, 139]. Nicht zu unterschätzen ist der intraoperative Verlust onkotisch wirksamer Moleküle (insbesondere Proteine) aus dem Intravasalraum, welcher einerseits durch den chirurgischen Blutverlust selbst, andererseits aber auch durch eine Verschiebung in das Interstitium („Protein-Shift“) bedingt ist [116, 117].

Die genannten Volumenangaben beziehen sich auf Eingriffe am offenen Bauch, es werden allerdings zunehmend auch für die großen Eingriffe der Viszeralchirurgie laparoskopische Techniken eingesetzt. Letztere unterscheiden sich durch weniger absolute Verluste z. B. aufgrund von Exenteration, hinzu kommen allerdings temporäre hämodynamische Effekte, wie eine Steigerung der Nachlast und des peripheren Widerstands sowie auch der Vorlast aufgrund des benötigten Kapnoperitoneums [9]. Außerdem werden viele dieser Eingriffe in extremen Lagerungen durchgeführt, welche zusätzlich zu relativen Volumenverschiebungen führen und beim Flüssigkeitsmanagement berücksichtigt werden müssen.

Der Flüssigkeitshaushalt kann auch postoperativ durch Nebenwirkungen der Anästhesie wie beispielsweise postoperative Übelkeit und Erbrechen oder chirurgische Komplikationen, wie z. B. Nachblutungen, Infektionen oder Fieber, beeinträchtigt sein. Auch Faktoren wie mangelnder Durst und/oder Appetit durch Bettlägerigkeit, eine einliegende Magensonde oder Medikamentennebenwirkungen wirken sich auf den Flüssigkeitshaushalt aus.

## Störungen des Flüssigkeitshaushaltes

**Figure 3:**
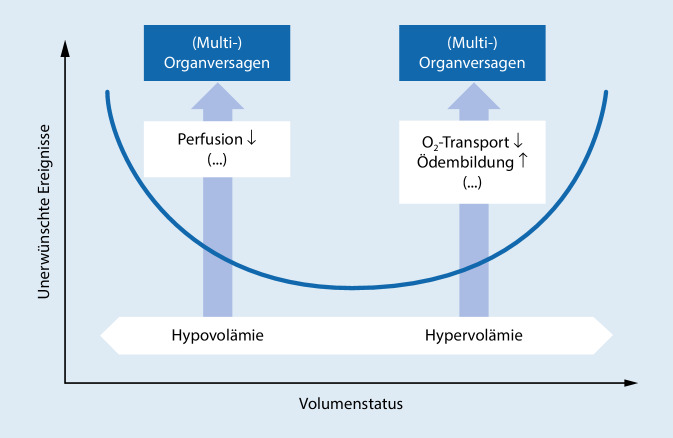

Sowohl zu wenig als auch zu viel intravasale Flüssigkeit wirken sich negativ auf das *Outcome* der Patienten aus. Die Beziehung zwischen applizierter Flüssigkeitsmenge und den damit verbundenen Komplikationen wurde bereits mehrfach mit einem U‑förmigen Verlauf dargestellt (Abb. 3; [11, 89, 130]).

Eine große retrospektive Datenanalyse zur perioperativen Flüssigkeitsgabe ergab, dass sich in der Summe der verschiedenen Endpunkte (30-Tage-Mortalität, Nierenversagen, Krankenhausverweildauer, respiratorische Komplikationen und Aufenthaltskosten) eine moderate intraoperative Flüssigkeitsgabe zwischen 900 und 1750 ml durchschnittlich am günstigsten für die Patienten auswirkte. Es ist jedoch zu berücksichtigen, dass es sich um ein gemischtes Kollektiv ohne nennenswerte Blutverluste (durchschnittlich <200 ml) handelte [126].

### Folgen für die Organfunktion

Zu den ernsten perioperativen Komplikationen, die mit Hypovolämie, Hypotonie und einer resultierenden Minderperfusion assoziiert sind sowie mit einer erhöhten Krankenhausverweildauer und einem Anstieg der Sterblichkeit einhergehen, zählt das akute Nierenversagen [60, 61]. Dieses kann umgekehrt jedoch auch als Folge hypervolämischer Zustände auftreten, wobei der hierdurch potenziell ausgelösten venösen Stauung eine besondere pathophysiologische Bedeutung zukommt. So führt ein erhöhter zentraler Venendruck (ZVD) infolge einer Flüssigkeitsüberladung zu einem venösen und interstitiellen Druckanstieg mit entsprechenden Konsequenzen für Perfusion, Mikrozirkulation und damit auch Funktion vorgeschalteter Organe [111]. In Bezug auf die Mikrozirkulation als Determinante der Sauerstoffversorgung und damit auch der Organfunktion wird insbesondere der Barrierefunktion der endothelialen Glykokalyx ein besonderer Stellenwert zugeschrieben. Tierexperimentelle Studien weisen darauf hin, dass ein Verlust der Glykokalyxintegrität mit einer gesteigerten Gefäßpermeabilität und der Gefahr einer interstitiellen Ödembildung einhergeht [48]. Inwiefern die perioperative Volumengabe die Barrierefunktion der Glykokalyx beeinträchtigt, wurde bislang noch nicht direkt untersucht. In einer prospektiven Studie an kardial gesunden Patienten, welche sich einem elektiven chirurgischen Eingriff unterzogen, konnten Chappell et al. jedoch zeigen, dass die Gabe von 20 ml/kgKG Hydroxethylstärke (HES) 130/0,4 mit erhöhten Serumwerten der Glykokalyxbestandteile Hyaluronan und Syndecan‑1 einhergeht [22]. Neben erhöhten intravasalen Drücken wird die Zerstörung der Glykokalyx mit konsekutivem Kapillarleck und entsprechenden Flüssigkeitsverschiebungen daher ebenfalls als möglicher Pathomechanismus des hypervolämieassoziierten Nierenversagens in Betracht gezogen [101].

Neben der Niere sind als Organe sowohl das Herz als auch die Lunge – insbesondere bei vorbestehender Schädigung – auf eine ausgeglichene Flüssigkeitsbilanz angewiesen. In einer Arbeit von Holte et al. hatten Patienten mit Darmoperationen bei restriktiver (1640 ml [935–2250] vs. 5050 ml [3563–8050]) Flüssigkeitsgabe 6 h postoperativ eine signifikant bessere Einsekundenkapazität und eine bessere Sauerstoffsättigung als in der liberalen Gruppe [43]. Außerdem konnten Casado et al. bei Ösophagektomien respiratorische Komplikationen sowie einen verlängerten Intensiv- (4,75 ± 1,21 vs. 1,02 ± 0,2 Tage) und Krankenhausaufenthalt (27,32 ± 8,23 vs. 14,12 ± 3,75 Tage) mit einer signifikant höheren Flüssigkeitsmenge (5415 ± 810 ml vs. 4174 ± 1033 ml, *p* < 0,01) assoziieren [20].

### Folgen für das operative Behandlungsergebnis

Exzessive Flüssigkeitssubstitution beeinflusst nachweislich das chirurgische Behandlungsergebnis. So wurde sowohl im Tiermodell als auch in retrospektiven Analysen festgestellt, dass diese zu signifikant weniger belastbaren Anastomosen führt [15, 82, 121]. Als Ursache wird einerseits eine ödematöse Schwellung sowie andererseits eine lokale Entzündungsreaktion mit konsekutiver Leukozytenimmigration angenommen [63]. Möglicherweise ist insbesondere die Zunahme des Körpergewichts ein guter Surrogatparameter für die Zunahme an Komplikationen, da gezeigt wurde, dass mit >10 %iger, aber insbesondere >20 %iger Steigerung des Körpergewichts die Mortalität signifikant zunimmt [21, 76]. Eine Gewichtszunahme von mehr als 3 kgKG nach einer Kolonoperation ist zudem mit einer Verlängerung des Krankenhausaufenthalts und einer verzögerten Magen-Darm-Passage assoziiert [74]. Umgekehrt hat sich mehrfach gezeigt, dass eine reduzierte intraoperative Volumenzufuhr mit einer besseren zellulären Immunität und einer geringeren Rate an Wundinfektion assoziiert werden kann [53, 143]. Eine 2018 in *New England Journal of Medicine *publizierte Studie zeigte hingegen auch bei restriktiver Flüssigkeitstherapie eine tendenziell höhere Rate an Sepsis und Wundinfektionen, ein weiterer Hinweis auf den genannten parabelförmigen Verlauf (Abb. 3; [93]).

## Perioperatives Flüssigkeitsmanagement

### Präoperative Phase

Als Gesamtziel des perioperativen Flüssigkeitsmanagements sollte nach aktuellem Kenntnisstand eine ausgeglichene Flüssigkeitsbilanz angestrebt werden. Zwar gilt in der präoperativen Phase eine leitliniengerechte Minimierung des Aspirationsrisikos mit Einhaltung der vorgeschriebenen Nüchternheit von 6 h für Nahrungsmittel und 2 h für klare Flüssigkeiten, allerdings sollten die angegebenen Intervalle nicht unnötig überschritten werden [5, 128]. Stress, erhöhter Leidensdruck der Patienten als auch negative Beeinträchtigungen im Wasser‑/Energiehaushalt können die Folge sein. In verschiedenen Leitlinien zu *Fast-Track*-Konzepten wird deshalb bei elektiven Eingriffen 2–3 h präoperativ die Gabe von 400 ml einer Zubereitung mit komplexen Kohlenhydraten (12,5 % Maltodextrin) empfohlen [37, 67, 88, 100]. Es konnte durch den Konsum von 800 ml am Vorabend und 400 ml des Getränks 2–3 h vor dem Eingriff eine erhaltene Insulinsensitivität, verbunden mit einem geringeren Verlust an Muskelmasse, gezeigt werden [99, 144]. Das Vermeiden einer Sarkopenie ist insbesondere für Patienten mit neoplastischen Erkrankungen und einem damit verbundenen protrahierten Krankheitsverlauf relevant. Eine geringere perioperative Morbidität sowie Mortalität wurden in einigen Veröffentlichungen u. a. für Kolon‑, Magen-, biliäre und Ösophagusoperationen beschrieben [45, 72, 103, 135]. Metaanalysen zu diesem Thema zeigen keine eindeutige Überlegenheit eines Einsatzes von komplexen Kohlenhydraten [13], es besteht aber Einigkeit, dass der Konsum der genannten Zubereitungen nicht zu verlängerten Magenentleerungszeiten oder anderen unerwünschten Effekten führt [40]. Die Maßnahme wirkt sich durch Verringerung von Angst, Durst und Hungergefühl jedoch positiv auf das Befinden der Patienten aus. Dies ist insofern relevant, da es im klinischen Alltag aufgrund von Notfällen und damit zeitlichen Verschiebungen im OP-Programm zu ungewollt längeren Nüchternheitsphasen kommen kann.

Die *Fast-Track-*Leitlinien raten zudem von einer regelhaften mechanischen Darmvorbereitung mit Laxanzien ab, da sowohl Wasser- und Elektrolythaushalt als auch Patientenkomfort beeinträchtigt werden. Umgekehrt konnten bei Unterlassen keine Nachteile bezüglich der Rate an Anastomoseninsuffizienzen, Reoperationen oder der Mortalität bei kolorektalen Eingriffen gezeigt werden [37, 67, 88, 100]. Durch eine adäquate Aufklärung und explizites Erlauben der oralen Flüssigkeitszufuhr im Rahmen der Empfehlungen kann bereits in der Prämedikationsambulanz maßgeblich zu einem erfolgreichen perioperativen Volumenmanagement beigetragen werden.

#### Merke

Operationsvorbereitung

- Nüchternheitszeiten über die empfohlenen Intervalle hinaus vermeiden (2 h für Flüssigkeiten, 6 h für feste Nahrung),
- präoperativ die orale Gabe von Zubereitungen mit komplexen Kohlenhydraten erwägen.

### Intraoperative Phase

#### Auswahl der geeigneten Überwachung

Zusätzlich zur klinischen Untersuchung empfiehlt die Leitlinie *Intravasale Volumentherapie beim Erwachsenen* apparative Parameter zur Bewertung des Flüssigkeitsstatus eines Patienten [83], wobei die Betrachtung isolierter Parameter nur wenig Aussagekraft besitzt. Eine intraoperative Oligurie kann häufig durch Stress oder Art des Eingriffs erklärt werden und stellt keinen validen Indikator zur Beurteilung des Flüssigkeitshaushalts dar [26, 84]. Dass nicht zuletzt der definierte Schwellenwert von <0,5 ml/kgKG und h für eine Oligurie wahrscheinlich keine direkte Relevanz für das Behandlungsergebnis hat, konnte in einer aktuellen retrospektiven Datenanalyse gezeigt werden [90]. In dieser Arbeit lag der „Cut-off“-Wert der intraoperativen Urinproduktion, unterhalb dessen das Risiko für ein akutes Nierenversagen signifikant ansteigt, bei 0,3 ml/kgKG und h.

Im Alltag stehen routinemäßig Parameter wie Herzfrequenz und Blutdruck zur Verfügung, welche ergänzend zur klinischen Einschätzung verwendet werden. Ein weiterer ist die Rekapillarisierungszeit, welche eine grobe Einschätzung von peripherer Durchblutung sowie der Mikrozirkulation erlaubt [52]. Als einfacher und schnell zu erhebender Wert kann diese in Zusammenschau mit anderen Parametern einen Zusatznutzen bieten. Die genannten klinischen Größen unterliegen jedoch einer Vielzahl von Einflussfaktoren und reagieren erst bei einer deutlichen Reduktion des Blutvolumens, was sie, einzeln betrachtet, zu ungenauen Indikatoren macht [129]. Auch die visuelle Einschätzung des intraoperativen Blutverlustes ist potenziell fehlerbehaftet und wird in einer kürzlich veröffentlichten Studie von einem Großteil der befragten Anästhesisten als unzureichend empfunden [109]. Durch mathematische Betrachtung des mittleren Hämatokrits sowie des Erythrozytenvolumens – wie von Rehm et al. beschrieben – besteht unter der Voraussetzung einer Normovolämie jedoch die Möglichkeit, den perioperativen Blutverlust zu berechnen [117].

Insbesondere bei größeren Eingriffen besteht oftmals auch die Indikation zur Anlage eines zentralen Venenkatheters, wodurch regelhaft auch der ZVD gemessen werden kann. Laut der aktuellen S3-Leitlinie *Intravasale Flüssigkeitstherapie beim Erwachsenen* sollte der ZVD perioperativ nicht zur Diagnose eines Volumenmangels herangezogen werden („grade of recommendation“ [GOR] A) [83]. Allerdings kann die ZVD-Dynamik im Verlauf der Operation durchaus auf eine gestörte Perfusion oder gar eine Hypervolämie hinweisen bzw. durch adäquate Einstellung, z. B. ZVD <5 mm Hg bei Leberoperationen, Blutverluste verhindern und damit zu einem erfolgreichen Volumenmanagement beitragen [10, 127]. Ähnliches gilt für die visuelle Abschätzung der Schlagvolumenvariabilität (SVV) anhand der arteriellen Druckkurve, welche auch ohne eine quantitative Auswertung Hinweise auf den Flüssigkeitsstatus liefern kann. Die Kombination der genannten Parameter reicht bei einer Großzahl von Eingriffen für eine Evaluation des Flüssigkeitsstatus bereits aus.

Je multimorbider der Patient und je größer der Eingriff, desto schwieriger und gleichzeitig auch entscheidender ist es, den Flüssigkeitsbedarf möglichst exakt zu bestimmen. In der Intensivmedizin etablierte Tests wie die sonographische Bestimmung der Atemvariabilität in der unteren Hohlvene oder der passive Beinhebetest sind im intraoperativen Kontext wenig geeignet bzw. oft nicht umsetzbar. Für den Pulmonalarterienkatheter konnten in der breiten Anwendung aufgrund der mit der Invasivität der Maßnahme verbundenen Komplikationen bisher keine Vorteile für die Patienten gezeigt werden, er sollte deshalb selektierten Patientenkollektiven vorbehalten werden [70]. Weitere in der Intensivmedizin eingesetzte Verfahren auf Basis von Thermo- oder Indikatordilution (z. B. PiCCO™, Getinge Deutschland GmbH, Rastatt, Deutschland) erfordern einen höheren apparativen Aufwand, während algorithmenbasierte Systeme ohne Indikatormessung (z. B. LIDCO™, LIDCO, London, UK) bisher noch nicht ausreichend validiert sind.

Die Erhebung dynamischer Parameter wie der Pulsdruck- und der Schlagvolumenvariabilität rückt jedoch zunehmend in den Vordergrund. Für deren Messung müssen allerdings Grundvoraussetzungen wie ein Sinusrhythmus ohne arrhythmische Episoden sowie eine invasive Beatmung mit Tidalvolumina >6 ml/kgKG erfüllt sein, wodurch bestimmte Patientenkollektive ausgeschlossen werden [139]. Bereits sehr gut validiert sind auch der transösophageale Dopplermonitor [1, 108] und die transösophageale Echokardiographie (TEE), welche jedoch untersucherabhängig ist. Hierbei ist zu beachten, dass im viszeralchirurgischen Bereich bestimmte Komorbiditäten (z. B. ausgeprägte Ösophagusvarizen) oder der geplante Eingriff *per se* eine Kontraindikation für die Einführung einer Sonde in den Ösophagus darstellen können.

Komplett nichtinvasive Verfahren wie der suprasternale Dopplerultraschall, die Radialarterientonometrie, die thorakale Bioimpedanz oder die Pulswellentransitzeit zeigen eine deutliche Streubreite bei den Messungen. Sie können daher nach Meinung von Pestel et al. die etablierten Referenzverfahren zum aktuellen Zeitpunkt nicht ersetzen [107].

In der Zusammenschau scheint eine Kombination aus klinischem Blick und den Parametern des (erweiterten) Basis-Monitoring (Elektrokardiogramm, Blutdruckmessung manuell/oszillatorisch, periphere Sauerstoffsättigung, invasive Blutdruckmessung, ZVD) für die meisten Eingriffe zur Beurteilung des Flüssigkeitshaushaltes auszureichen. Dieses muss bei Hochrisikopatienten ggf. um weitere, invasivere Messmethoden ergänzt werden.

#### Eigenschaften kristalloider und kolloidaler Lösungen

Entscheidend für die i.v.-Therapie sind nicht nur Menge, Zeitpunkt und Applikationsweg, sondern auch die Zusammensetzung der Flüssigkeit selbst. Am häufigsten sind nahezu isotonische Lösungen im Gebrauch. Das wahrscheinlich bekannteste Präparat ist 0,9 %ige Natriumchlorid(NaCl)-Lösung (leicht hyperton), welche zu gleichen Teilen aus je 154 mmol/l Natrium und Chlorid besteht [115]. Daneben gibt es sog. balancierte Vollelektrolytlösungen (Ringer-Lösung®, Plasmalyte® oder Sterofundin®), welche den physiologischen Elektrolytkonzentration im menschlichen Blut ähneln (angepasste Chloridkonzentration, Zusatz von organischen Anionen wie Lactat, Acetat oder Malat sowie weiterer Elektrolyte wie Kalium, Magnesium oder Kalzium in unterschiedlichen Konzentrationen [115]). Die Vor- und Nachteile der unterschiedlichen Präparate in der klinischen Praxis werden im Anschluss dargelegt.

Zu den wichtigsten Limitationen kristalloider Lösungen gehört deren Eigenschaft der freien Diffusion, d. h. die Möglichkeit, auch die intakte Gefäßbarriere zu überwinden. So wurde gezeigt, dass abhängig von der Intaktheit der Gefäßbarriere und dem Flüssigkeits‑/Volumenstatus des Patienten teilweise bereits innerhalb der ersten Stunde nach Applikation lediglich 20 % des infundierten Volumens im Gefäßsystem verbleiben, während der Rest in das Interstitium diffundiert [50]. Sollen beispielsweise Blut- oder anderweitige Flüssigkeitsverluste ausgeglichen werden, wird häufig ein Ersatz des Blutverlustes im Verhältnis 3:1 mit Kristalloiden empfohlen [68, 117]. Als Konsequenz kann eine „Überwässerung“ mit Flüssigkeitsverschiebungen und den bereits erwähnten Nebenwirkungen resultieren. Dies ist sicherlich einer der Gründe, bei größeren Flüssigkeitsverlusten ergänzend zu Kristalloiden auf weitere Alternativen wie Kolloide zurückzugreifen. Der Volumeneffekt scheint kontextsensitiv zu sein und ist ausgeprägter bei bereits bestehendem Volumenmangel im Vergleich zur prophylaktischen Kolloidgabe [51]. Unter optimalen Bedingungen wird hierdurch initial ein Volumeneffekt von annähernd 100 % der infundierten Menge erreicht [23, 47, 50].

Bei den Kolloiden werden synthetische von nichtsynthetischen (natürlichen) Präparaten unterschieden. Synthetische Kolloide basieren auf langkettigen Zuckermolekülen (z. B. HES, Dextrane) oder Proteinen (z. B. Gelatine). Darüber hinaus unterscheiden sich die genannten Lösungen im Hinblick auf Konzentration (z. B. HES 6 % oder 10 %) sowie Größe der enthaltenen Moleküle (z. B. 450.000 Dalton in der ersten (HES 450/0,7) und 130.000 Dalton (HES 130/0,4) in der dritten Generation). Anfangs basierten Kolloide auf unbalancierten Lösungen, wurden aber weiterentwickelt und stehen heute auch als balancierte Lösungen zur Verfügung. Insbesondere stärkehaltige Präparate sind auch durch den Nachweis einer höheren Mortalität und einer höheren Inzidenz von Nierenersatzverfahren bei septischen Patienten nach HES-Therapie in die Kritik geraten [38, 106, 145]. Weitere Nebenwirkungen der stärkehaltigen Präparate sind u. a. Juckreiz sowie dosisabhängige Störungen der Blutgerinnung [73, 124]. Im Juni 2013 wurde durch das Bundesinstitut für Arzneimittel und Medizinprodukte deshalb die Empfehlung herausgegeben, „von der Anwendung hydroxyethylstärkehaltiger Infusionslösungen abzusehen“. In der Folge publizierte Metaanalysen konnten im Bereich der Intensivtherapie eine erhöhte Rate an Nierenversagen nach Kolloideinsatz bestätigen [38, 145].

Nach einer Risikobewertung wird von einer Therapie mit HES bei Patienten mit Niereninsuffizienz, Sepsis und schweren Verbrennungen abgeraten [27]. Seit 2019 ist für die Verwendung von HES-haltigen Infusionslösungen eine *Online*-Schulung mit einen erfolgreich bestandenen *Multiple-Choice*-Test erforderlich. Jeder Anwender muss somit ein Zertifikat, „HES-Führerschein“, vorweisen, um das Präparat weiterhin bei Patienten anwenden zu dürfen (Infobox 1).

##### Infobox 1 Überblick über das HES-Schulungszertifikat

- Erwerb über die ESA-Akademie, Website: https://academy.esahq.org/esa/
- Online-Schulung mit 4 Modulen + Multiple-Choice-Test mit 4 Fragen
- Verpflichtend für alle Anwender seit April 2019, damit die Institution weiter mit HES beliefert werden kann
- Jährliche Wiederholung der Schulung
- Anwendung von HES-haltigen Infusionslösungen
  *Indikationen*: Volumenersatz bei Hypovolämie im Rahmen einer akuten Blutung, wenn kristalloide Lösungen nicht ausreichend sind
  *Kontraindikationen*: Kritisch kranke Patienten, Sepsis, Nierenschädigung oder Nierenersatztherapie
  *Weitere*: Überempfindlichkeit gegen die Wirkstoffe, Verbrennung, Dehydratation, Hyperhydratation, Lungenödem, intrakranielle/intrazerebrale Blutungen, organtransplantierte Patienten, schwere Gerinnungsstörungen, dekompensierte Herzinsuffizienz, schwere Elektrolytentgleisungen, schwere Leberfunktionsstörungen
  *Dosis und Dauer der Anwendung*: geringst mögliche Dosis verwenden; Höchstdosis beachten (30 ml/kgKG); Anwendung für max. 24 h unter hämodynamischem Monitoring
  *Warnhinweise*:
  - Eine Volumenüberladung sollte vermieden werden
  - Eine Kontrolle der Nierenfunktion wird für 90 Tage empfohlen
  - Bei Anzeichen einer Verschlechterung der Nierenfunktion sollte der Einsatz von HES beendet werden

Trotz dieser Kontraindikationen für HES schien das Produkt bis zuletzt insbesondere beim Volumenmangelschock nach Trauma und im perioperativen Setting durchaus noch seine Berechtigung zu haben.

Ein kürzlich erschienener Wissenschaftsreport aus dem deutschen Traumaregister wirft allerdings auch für die bis dato als sicher erachtete Indikation der HES-Gabe bei hypovolämischem Schock neue Fragen auf [41]. Die Autoren konnten nach einer Analyse von über 48.000 Patienten zwischen 2002 und 2015 einen Zusammenhang zwischen der Therapie mit >1000 ml an synthetischen Kolloiden und einer erhöhten Rate an Nierenersatzverfahren sowie Multiorganversagen feststellen. Für diese Indikation wird deshalb aktuell die prospektive THETHYS (*PragmaTic, prospEctive, randomized, controlled, doubleblind, mulTicentre, multinational study on the safety and efficacy of a 6* *% HydroxYethyl Starch (HES) solution versus an electrolyte solution in trauma patients*) durchgeführt. Diese untersucht als primäre Endpunkte das Überleben und die Beeinträchtigung der Nierenfunktion nach HES-Gabe bei Traumapatienten; ob sich die negativen HES-Effekte aus den Daten des Traumaregisters prospektiv bestätigen, bleibt bis zur Veröffentlichung der Ergebnisse die spannende Frage (https://www.esahq.org/~/media/ESA/Files/ClinicalTrialNetwork/CRO/HES%20TETHYS%20%20Flyer%20v21%2023AUG16.ashx).

Gelatinehaltige Präparate werden in Deutschland aktuell überwiegend als 4 %ige Lösung vertrieben. Es stehen sowohl ein Präparat auf NaCl-Basis als auch eine balancierte Form zur Verfügung. Der Einsatz erfolgt in der Regel gleichwertig zu den stärkehaltigen Infusionslösungen bei akutem Volumenmangel. Über das Sicherheitsprofil von Gelatine lässt sich jedoch nur bedingt eine Aussage treffen, wobei die unerwünschten Effekte auf die Gerinnung und die Nierenfunktion den HES-Präparaten ähnlich zu sein scheinen. Gelatinelösungen sind zudem mit einer höheren Inzidenz von z. T. erheblichen anaphylaktoiden Reaktionen assoziiert [91].

Als natürliche Kolloide gelten u. a. Humanalbumin in verschiedenen Konzentrationen sowie auch Gefrierplasma, wobei Letzteres keine Indikation zum primären Volumenersatz hat (Klassifizierung: 1C+) [19]. Humanalbumin wird aus Plasma hergestellt und in verschiedenen Konzentrationen z. B. 5 %ig oder 20 %ig vertrieben. Es gilt als nicht immunogen und ist mit Blick auf die aktuelle Studienlage gut verträglich. Schwerwiegende Nebenwirkungen wurden bisher nicht beschrieben [118]. Eine Übersicht der aktuell zur Verfügung stehenden Kolloide bietet Tab. 1.

**Table 1:** 

	Volumeneffekt	Initiale Halbwertszeit	Kolloidosmotischer Druck	Molekülgröße	Balanciert verfügbar?	Maximaldosis	Besonderheiten
*HES 130/0,4*	98 ± 12 %	Ca. 4–6 h	25–35 mm Hg	Ca. 130.000 Dalton	Ja	30 ml/kgKG	s. Infobox 1
*HES 200/0,5*	90 ± 18 %	Ca. 4–6 h	25–35 mm Hg	Ca. 200.000 Dalton	Ja	30 ml/kgKG	s. Infobox 1
*Gelatine, 4* *%*	50–100 %	Ca. 5–8 h	25–35 mm Hg	Ca. 30.000 Dalton	Ja	Je nach Hämodilution	Anaphylaktische Reaktion
*Albumin, 5* *%*	87 ± 14 %	Ca. 19 Tage	25–35 mm Hg	Ca. 66.000 Dalton	„Salzarm“ (Na^+^ 125 mmol/l Cl^−^ 100 mmol/l)	Je nach Volumenmangel und Proteinverlust	–
*Albumin, 20* *%*	185 ± 63 %	Ca. 19 Tage	80–120 mm Hg	Ca. 66.000 Dalton	„Salzarm“ (Na^+^ 125 mmol/l Cl^−^ 100 mmol/l)	Je nach Volumenmangel und Proteinverlust	–

*HES* Hydroxyethylstärke, *Na*^*+*^ Natriumionen , *Cl*^*–*^ Chloridionen

#### Vergleichende Studien

Beim Vergleich der verschiedenen Kristalloide zeigte eine Metaanalyse von 21 Studien mit insgesamt ca. 6200 Patienten, welche den Einsatz von Kochsalzlösung mit balancierten Lösungen verglichen haben, dass es schwache Evidenz für eine höhere Morbiditätsrate (u. a. für metabolische Acidose, die Notwendigkeit einer Bluttransfusion und einer Hyperchloridämie) unter Therapie mit NaCl 0,9 % gibt. Dahingegen unterscheidet sich die Letalität im Gruppenvergleich nicht signifikant [62]. Auch neuere Vergleichsstudien zeigen bei den perioperativen Komplikationen keine Überlegenheit für NaCl 0,9 % oder balancierte Kristalloide [80]. Dennoch konnten 2 in *New England Journal of Medicine *erschienene Studien, eine in der Notaufnahme und eine auf Intensivstationen, zeigen, dass der Einsatz von balancierten Kristalloiden mit einer signifikant niedrigeren Inzidenz von akutem Nierenversagen einhergeht [122, 123]. Zudem zeigte sich bei den kritisch kranken Patienten eine Tendenz zu einer niedrigeren 30-Tage-Sterblichkeit in der Gruppe der balancierten Kristalloide [123].

Der Einsatz von balancierten Kristalloiden wird in der S3-Leitlinie *Intravasale Volumentherapie beim Erwachsenen* aktuell als Mittel der Wahl im periinterventionellen Kontext empfohlen [83]. Trotz dieses Konsenses zugunsten der balancierten Lösungen gibt es jedoch bisher keine klare Evidenz, welche der verschiedenen Zusammensetzungen – beispielsweise auf Basis von Lactat oder Acetat – ideal ist.

Für den Vergleich zu balancierten Lösungen existiert zudem eine Cochrane-Metaanalyse mit knapp 10.000 Patienten für den Vergleich mit Albumin, etwa 9000 eingeschlossenen Patienten für den Vergleich mit HES sowie 11 Studien zu Gelatine und 9 Studien zu Dextranpräparaten. Für keines der genannten Kolloidpräparate konnte ein Vorteil gegenüber kristalloiden Lösungen hinsichtlich der Mortalität in Trauma‑, Verbrennungs- und operierten Patienten gezeigt werden; bei HES ist sogar eine Erhöhung der Sterblichkeit nicht auszuschließen [105].

Beim Flüssigkeitsersatz in der elektiven Chirurgie kann aufgrund der aktuell vorliegenden Datenlage zwar keine Überlegenheit für Kristalloide oder HES gezeigt werden, dennoch gibt es eine Vielzahl von Hinweisen auf negative Effekte der HES-Gabe auch im operativen Kontext [30, 113]. Eine Metaanalyse von Wilkes et al. aus dem Jahr 2014, welche annähernd 4500 allgemein- und kardiochirurgische Patienten aus insgesamt 15 Studien untersucht, zeigt jedoch unabhängig vom Eingriff ein erhöhtes Risiko für den postoperativen Bedarf von Nierenersatzverfahren nach intraoperativer Verabreichung von HES [77]. Dahingegen konnte eine kürzlich erschienene retrospektive Analyse keine negativen Effekte einer perioperativen HES-Gabe auf Nierenfunktion, Mortalität oder Krankenhausverweildauer feststellen [102]. Eine weitere prospektive, randomisierte multizentrische Studie, PHOENICS (*Safety and Efficacy of 6 % Hydroxyethyl Starch (HES) Solution Versus an Electrolyte Solution in Patients Undergoing Elective Abdominal Surgery*), welche den Einsatz von HES im Vergleich mit balancierten Kristalloiden bei elektiver Abdominalchirurgie untersucht, verspricht, hierfür neue Erkenntnisse zu liefern. Primärer Endpunkt der Studie ist eine Veränderung der glomerulären Filtrationsrate mittels Cystatin-C-Bestimmung. Zusätzlich werden u. a. Endpunkte wie die postoperative Komplikationsrate und die Sterblichkeit innerhalb des ersten Jahres nach Operation untersucht. Eine Komplettierung der Studie wird noch im Jahr 2020 erwartet (https://clinicaltrials.gov/ct2/show/NCT03278548).

Auch der Einsatz möglicher HES-Alternativen ist nicht unumstritten. Gelatinehaltige Präparate dürfen zwar zur Therapie der akuten perioperativen Hypovolämie weiterhin gleichwertig mit HES und Kristalloiden genutzt werden (GOR 0, S3-Leitlinie, [83]). Die aktuelle Studienlage deutet aber darauf hin, dass eine Umstellung des Therapieregimes von HES auf gelatinehaltige Präparate nicht mit einer verringerten Rate an akutem Nierenversagen oder Mortalität einhergeht [4].

Für Albumin, welches in der *SAFE*-Studie 2004 im Vergleich mit Kochsalzlösung nicht mit vermehrten Komplikationen einherging, konnte im Vergleich zu Kristalloiden bislang keine eindeutige Überlegenheit gezeigt werden [105, 118]. Inwiefern der Einsatz von Alternativen, wie z. B. Humanalbumin, weniger risikobehaftet bzw. überlegen ist, wurde u. a. in der prospektiven *CHART-*Studie untersucht, wobei Humanalbumin 5 % mit HES 6 % (130/0,4) bei Patienten mit radikaler Zystektomie verglichen wurde. Als Endpunkt steht in erster Linie Cystatin C als Parameter für die Nierenfunktion bis 90 Tage nach der Operation im Vordergrund, aber auch sekundäre Endpunkte wie der Einfluss auf die Glykokalyx und die Länge des Intensiv- und Krankenhausaufenthaltes wurden untersucht [57]. Bezüglich der ausgewerteten Endpunkte zeigte sich ein vergleichbares Sicherheitsprofil für Albumin 5 % und HES 6 % [57]. Eine Analyse von zielgerichteter Therapie mit Albumin 5 % im Vergleich zu Ringer-Acetat-Lösung bei großen viszeralchirurgischen Eingriffen zeigte keine Überlegenheit bei dem primären Endpunkt Sauerstoffangebot oder bei den sekundären Endpunkten z. B. verschiedenen hämodynamischen Variablen und Krankenhausverweildauer [14]. Für den Einsatz von hypertonen Salzlösungen kann aktuell aufgrund der Datenlage noch keine eindeutige Aussage getroffen werden [85].

#### Zusätzliche Maßnahmen

Insbesondere relativen Volumenverschiebungen, welche u. a. durch Vasodilatation als Anästhesienebenwirkung, durch den Einsatz eines Kapnoperitoneums oder intraoperative Lagerungsmanöver auftreten, muss nicht zwangsweise mit Volumen begegnet werden. Der mäßige Einsatz von Vasopressoren, allen voran Noradrenalin, scheint geeignet, um den Blutdruck aufrechtzuerhalten und die genannten temporären Änderungen zu kompensieren [141]. Bei radikalen Zystektomien wurden im Vergleich zur Kontrollgruppe (Ergänzung durch Noradrenalin 2–8 µg/kgKG und h vs. alleinige Flüssigkeitsgabe) signifikant weniger gastrointestinale sowie kardiale Komplikationen festgestellt (6 % vs. 37 %, *p* < 0,0001). Umgekehrt scheint es auch einen Zusammenhang zwischen Katecholamingabe und Anastomoseninsuffizienzen zu geben; Letztere waren bei Katecholamintherapie bis zu 4‑fach erhöht, wie in einer Arbeit zur *damage control surgery* gezeigt wurde [28]. Die kontinuierliche Gabe von Katecholaminen sollte somit vigilant erfolgen und muss auch bei der Bewertung anderer Parameter des Flüssigkeitshaushalts Berücksichtigung finden. Es wurde z. B. berichtet, dass die Gabe von Phenylephrin bei Leberoperationen zu einer erhöhten Flüssigkeitsreagibilität führte [95]. Die komplexen Wirkmechanismen der Katecholamine z. B. auf Vor- und Nachlast könnten somit zu einer Fehleinschätzung des Flüssigkeitsstatus führen.

Zusammenfassend scheint momentan eine Erhaltungstherapie auf Basis von balancierten Kristalloiden – ggf. ergänzt durch Vasopressoren – sinnvoll, während der Einsatz von synthetischen Kolloiden streng abgewogen werden muss und die Gabe von Albumin zwar sicher scheint, es aber keine Evidenz für eine Überlegenheit gegenüber Kristalloiden gibt.

#### Liberale, restriktive bzw. nullbilanzierte und zielgerichtete Therapiekonzepte

Die intraoperative Flüssigkeitstherapie hat sich in den letzten Jahren stark gewandelt. Während noch in den 1980er-Jahren für elektive Operationen großzügig mehrere Liter Flüssigkeit infundiert wurden, haben neuere Daten zur i.v.-Flüssigkeitstherapie dieses Regime infrage gestellt [18]. Zurzeit gibt es im Wesentlichen 3 verschiedene Ansätze zur Volumentherapie bei größeren viszeralchirurgischen Eingriffen, welche man als liberal, restriktiv bzw. nullbilanziert („zero balance“) oder zielgerichtet (GDT) bezeichnen kann.

Die Entwicklung verschiedener *Fast-Track*-Programme seit Ende der 1990er-Jahre rückte eine bilanzierte Flüssigkeitstherapie auch in der Viszeralchirurgie als Puzzlestein eines multimodalen Konzeptes zunehmend in den Vordergrund [58]. Mit *Fast Track* wird ein Vorgehen bezeichnet, welches durch Optimierung der perioperativen Umstände z. B. keine Drainagen, frühes Entfernen der Magensonde und Reduktion der i.v.-Flüssigkeitstherapie durch Oralisierung der Flüssigkeitszufuhr eine schnellstmögliche Genesung der Patienten sowie eine minimal mögliche Krankenhausverweildauer forciert [140]. Als Vorreiter für dieses Therapiekonzept gilt die kolorektale Chirurgie, in welcher eine solche Vorgehensweise mittlerweile gängiger Standard ist [37]. Außerdem scheinen *Fast-Track*-Protokolle auch in der Pankreaschirurgie mit einer besseren Versorgungsqualität assoziiert zu sein, ohne dabei die Patientensicherheit zu gefährden [55]. Anfänglich konnte zwar kein Zusammenhang zwischen applizierter Flüssigkeitsmenge und perioperativer Morbidität hergestellt werden [36, 87]. Neuen Veröffentlichungen zufolge profitieren auch Patienten mit Eingriffen am Pankreas durch verringerte Komplikationsraten (Wundinfektionen und Revisionseingriffe) sowie eine kürzere Krankenhausverweildauer von restriktiver Flüssigkeitsgabe [64, 69, 137, 138]. Der Zusammenhang zwischen Komplikationen und applizierter Flüssigkeit scheint aber auch in der Pankreaschirurgie der bereits beschriebenen U‑Form zu folgen [6, 119].

Es gibt auch Untersuchungen zu *Fast-Track*-Konzepten mit zurückhaltender Flüssigkeitsgabe im Rahmen von Leberresektionen und Operationen am oberen Gastrointestinaltrakt, wobei Vorteile hinsichtlich postoperativer Komplikationen (Leberversagen, Aszites und Krankenhausverweildauer) gegenüber dem konventionellen Vorgehen beschrieben wurden [17, 114]. Insbesondere bei Ösophagektomien ging eine exzessive Flüssigkeitstherapie mit einer erhöhten Rate an Komplikationen einher [20, 34].

Aufgrund der unterschiedlichen Definitionen von restriktiven und liberalen Flüssigkeitsregimen in einzelnen Studien stellt eine einheitliche Nomenklatur die Basis dar, um verschiedene Studien miteinander zu vergleichen. So wurde in einer Metaanalyse von Rahbari et al. für alle eingeschlossenen Studien der Flüssigkeitsbedarf mittels einer Formel berechnet und mit dem tatsächlich applizierten Volumen verglichen. Gruppen, die 10 % weniger als die berechnete Standardmenge erhielten, galten als „restriktiv“, die anderen wurden als „supplemental“ (ergänzend) klassifiziert. Insgesamt waren v. a. Koloneingriffe vertreten, und es konnten Vorteile einer restriktiven Flüssigkeitsgabe in Bezug auf postoperative Komplikationen nachgewiesen werden [112]. Vergleichbare positive Ergebnisse zugunsten von restriktiver oder zielgerichteter gegenüber einer liberalen Volumentherapie konnten auch für andere große viszeralchirurgische Eingriffe wie beispielsweise Magen- oder Pankreasoperationen gezeigt werden [3, 25].

Allerdings zeigte eine weitere Metaanalyse mit 7 Studien bei überwiegend kolorektalen Operationen im Gegensatz zu der von Rahbari et al. keine signifikanten Vorteile für eine restriktive Flüssigkeitstherapie, obwohl 2 der 7 Studien und explizit ähnliche Einschlusskriterien in beiden Metaanalysen berücksichtigt wurden [16]. Die Autoren einer weiteren Arbeit kommen zu dem Ergebnis, dass die Patienten mit einem normovolämischen Flüssigkeitsstatus eine geringere Wahrscheinlichkeit für postoperative Komplikationen hatten [134]. Interessanterweise wurde bei vielen der eingeschlossenen Studien die restriktive Patientengruppe durch die Autoren als normovolämisch klassifiziert, womit letztendlich ein ähnliches Ergebnis wie in der Publikation von Rahbari et al. skizziert wird.

Eine Übersicht von prospektiven Studien und deren Fazit seit der ersten Publikation durch Brandstrup et al. [18] zeigt Tab. 2. Als bisher größte Analyse zum Vergleich von restriktiver und liberaler Flüssigkeitstherapie gilt die 2018 im *New England Journal of Medicine* veröffentlichte RELIEF-Studie [93]. Hierbei handelt es sich um eine randomisierte multizentrische Studie mit knapp 3000 Patienten, von denen über 60 % mindestens als ASA III klassifiziert wurden. Als primärer Endpunkt wurden das Einjahresüberleben ohne nennenswerte Einschränkungen sowie sekundär diverse perioperative Komplikationen untersucht. Basis der Flüssigkeitstherapie waren kristalloide Lösungen mit einer Rate von 8 ml/kgKG und h in der liberalen und 5 ml/kgKG und h in der restriktiven Gruppe, außerdem wurde zur Narkoseeinleitung ein Bolus von 10 bzw. <5 ml/kgKG verabreicht. In der restriktiven Flüssigkeitsgruppe wurde während der Operation im Median etwa halb so viel Flüssigkeit verabreicht wie in der liberalen Gruppe (1677 ml vs. 3000 ml Kristalloide + je 500 ml Kolloid). Wider Erwarten konnten jedoch beim primären Endpunkt keine Vorteile für eine restriktive Flüssigkeitstherapie gezeigt werden. Zudem zeigt sich bei den sekundären Endpunkten (Wundinfektionen, Sepsis und Einsatz von Nierenersatzverfahren) ein Trend zu mehr Komplikationen in der restriktiven Gruppe. Die Autoren der Arbeit folgern daraus, dass eine moderat liberale Flüssigkeitstherapie im Hinblick auf perioperative Morbidität sicherer sein könnte als ein streng restriktives Regime.

**Table 2:** 

Studie [Zeitpunkt]	Art der Operation	Pat.-Zahl *n* =	Operationsdauer (min)	Blutverlust (ml)	Flüssigkeitsmenge intraoperativ (ml)	Art der Flüssigkeit	Fazit bei restriktiver Flüssigkeitstherapie
Brandstrup et al. (2003) [18] *[Intra-* *+* *postop.]* *Median* *+* *range*	Kolorektal	R: 69 S: 72	R: 180 (90–360) S**: **180 (120–390)	R: 400 (0–4530) S: 500 (0–1600)	R: 2740 (1100–8050) S: 5388 (2700–11.083) [Op.-Tag]	NaCl 0,9 %, Glucose 0,5 %, HES 6 % …	Weniger postoperative Komplikationen
**Kabon et al. (2005)** [54] *[Intraop.]*	Kolon	**R: **124 **L: **129	**R: **156 ± 66 **L: **156 ± 60	**R:** 333 ± 349 **L:** 322 ± 331	**R:** 2500 ± 1300 **L:** 3900 ± 1900	RL	Kein Einfluss auf Wundinfektionsrisiko
Nisanevich et al. (2005) [98] *[Intraop.]*	Große Viszeralchirurgie	R: 77 L: 75	R: 268 ± 112 L: 251 ± 91	R: 400 (50–2100) L: 440 (50–1800)	R: 1230 (490–7810) L: 3670 (1880–8800)	RL + HES	Kürzere Krankenhausverweildauer, weniger postoperative Komplikationen
Holte et al. (2007) [44] *[Intra-* *+* *postop.]*	Kolon	R: 16 L: 16	R: 119 (77–198) L: 121 (88–182)	R: 200 (10–980) L: 305 (0–1600)	R: 1140 (580–1500); Kolloid: 500 (350–750) L: 3900 (2722–6500) Kolloid: 500 (341–850)	RL + Voluven®	Kurzfristig schlechtere Lungenfunktion, aber weniger Hypoxämien
De Aguilar-Nascimento et al. (2009) [3] *[Intra-* *+* *postop.]*	Große Viszeralchirurgie	R: 28 C: 33	R: 230 (105–430) C: 270 (110–510)	N. a.	R: 4400 ± 1600 C: 5400 ± 1900	Kristalloide	Kürzere Krankenhausverweildauer, weniger postoperative Komplikationen
McArdle et al. (2009) [86] *[Intra-* *+* *postop.]*	Elektives offenes Bauchaortenaneurysma	R: 10 S: 11	R: 163,8 ± 10,2 S: 141,5 ± 7,5	R: 1146 ± 242 S: 1100 ± 163	R: 2626 ± 478 S: 3309 ± 216	RL, NaCl 0,9 %	Kürzere Krankenhausverweildauer, weniger postoperative Komplikationen
Cohn et al. (2010) [24] *[Intraop.]*	Kolorektal	R: 18 S: 9	N. a.	N. a.	R: 1861 ± 1448 S: 3635 ± 1694	RL	Kein negativer Einfluss auf NIRS-Messung
Futier et al. (2010) [29] *[Intraop.]*	Große Viszeralchirurgie	R: 36 C: 34	R: 257 ± 66 C: 224 ± 72	R: 425 ± 398 C: 278 ± 298	R: 3040 ± 769; Kolloid: 854 ± 547 C: 5266 ± 1340; Kolloid: 316 ± 311	RL + HES 6 %	Erhöhte Rate an Hypovolämien und postoperativen Komplikationen
Lobo et al. (2011) [75] *[Intraop.]*	Gemischt (v. a. kolorektal)	R: 45 C: 43	R: 250 ± 60 C: 228 ± 53	N. a.	R: 2301 ± 1064; Kolloid: 1216 ± 814 S: 4335 ± 1546; Kolloid: 915 ± 559	RL + Gelatine	Bei gutem O_2_-Angebot geringere Rate an postop. Komplikationen
Abraham-Nordling et al. (2012) [2] *[Intra-* *+* *postop.]*	Kolorektal	R: 79 S: 82	N. a.	R: 100 (100–200) S: 100 (100–300)	R: 575 (452–800) S: 2500 (2000–3070)	Ringer-Acetat, gepufferte Glucose 2,5 %	Keine Reduktion der Krankenhausverweildauer, aber weniger Komplikationen
Gao et al. (2012) [31] *[Intra-* *+* *postop.]*	Große Viszeralchirurgie	R: 93 S: 86	R: 180 (72–438) S: 180 (150–480)	R: 350 (0–3700) S: 420 (0–5400)	R: 1320 ± 220; Kolloid: 210 ± 300 S: 1450 ± 310; Kolloid: 1240 ± 410 [Op.-Tag]	RL + HES 6 %	Bessere zelluläre Immunität und weniger postoperative Komplikationen
Kalyan et al. (2013) [56] *[Intra-* *+* *postop.]*	Oberer GI-Trakt und kolorektal	R: 118 L: 121	R: 161 (32–343) S: 145 (40–285)	R: 400 (50–4245) S: 403 (63–2500)	R: 1000 (690–1500) S: 2033 (1576–2500)	RL + HES	Kein Unterschiede bei Verweildauer, Komplikationen und Krankenhausmortalität
Lavu et al. (2014) [69] *[Intra-* *+* *postop.]*	Pankreas	H: 131 C: 128	H: 414 (265–686) C: 386 (247–674)	H: 350 (45–2250) C: 400 (50–6900)	H: 11,0 (2,8–26,5) C: 14,7 (4,7–45) [ml/kgKG und h]	RL + 3 % NaCl (1 ml/kgKG und h)	Moderate Restriktion führt zu weniger postoperativen Komplikationen
Grant et al. (2016) [35] *[Intra-* *+* *postop.]*	Pankreas	R: 166 L: 164	R: 206 (68–462) L: 197 (44–379)	N. a.	R: 2050 (650–5130); Kolloid: 390 (0–2500) L: 3563 (1050–7550); Kolloid: 363 (0–4000)	Kristalloide, Albumin 5 %	Keine signifikanten Unterschiede bei perioperativen Komplikationen
Van Samkar et al. (2015) [133] *[Intraop.]*	Pankreas	R: 26 S: 22	R: 289 (78) S: 248 (104)	R: 1100 ± 600 S: 1000 ± 800	R: 2000 (1400–2400); Kolloid: 1400 ± 600 S: 2500 (1700–3600); Kolloid: 1000 ± 600	RL + HES	Keine Unterschiede bei der Magenentleerungszeit
Myles et al. (2018) [93] *[Intra-* *+* *postop.]*	Gemischt abdominal	R: 1490 L: 1493	R: 198 (144–276) L: 198 (150–270)	R: 200 (100–400) L: 200 (100–500)	R: 1677 (1173–2294) Kolloid (*n* = 369): 500 (250–800) L: 3000 (2100–3850) Kolloide: (*n* = 309): 500 (400–1000)	V. a. balancierte Kristalloide, ggf. EK/Kolloide	Keine Vorteile für behinderungsfreies Überleben nach einem Jahr, erhöhte Rate an periop. Nierenversagen

*R* restriktiv, *L* liberal, *n.a.* nicht angegeben, *C* „conventional/conservative“, *S* Standard, *H* „hypertonic saline“, *RL* Ringer-Laktat, *HES* Hydroxyethylstärke

Zunehmend wird auch die GDT, d. h. eine an definierten Zielwerten orientierte Flüssigkeitstherapie proklamiert, welche sich an definierten Richtwerten, beispielsweise für die SVV orientiert. Mit der stetig wachsenden Zahl der minimal-invasiven Überwachungsverfahren kommt dieser Art der Therapiesteuerung eine neue Bedeutung zu. In die *OPTIMISE*-Studie von Pearse et al. wurden 734 Hochrisikopatienten im Alter über 50 Jahren eingeschlossen, die sich einem größeren Eingriff am Gastrointestinaltrakt unterzogen. Im Vordergrund stand eine Optimierung des Schlagvolumens mit Flüssigkeit und Inotropika nach einem festgelegten Algorithmus. Bei der Auswertung der beiden verglichenen Kohorten GDT vs. „standard care“ zeigten sich keine Vorteile für Komplikationen und 30-Tage-Überleben in einer der Gruppen [104]. Eine weitere Multizenterstudie (*POEMAS* [*PeriOperative goal-directed thErapy in Major Abdominal Surgery*]) mit 142 Patienten, die sich offenen Operationen am Gastrointestinaltrakt unterzogen, untersuchte den Effekt von nichtinvasivem „cardiac output monitoring“ auf die postoperative Komplikationsrate sowie die Krankenhausverweildauer. Beide Endpunkte unterschieden sich nicht signifikant in den untersuchten Gruppen. Des Weiteren wurde in der Standard- und der GDT-Gruppe durchschnittlich eine ähnliche Menge an Flüssigkeit appliziert, was den Nutzen des *Cardiac output monitoring* in Bezug auf Flüssigkeitstherapie weiter infrage stellen könnte. Es ist an dieser Stelle jedoch zu berücksichtigen, dass es sich bei beiden Studien um ein relativ gesundes Patientenkollektiv mit je etwa 50 % ASA-I- und ASA-II-Patienten handelte.

Mehrere andere Studien konnten Vorteile wie u. a. eine verringerte postoperative Morbidität und eine schnellere Erholung der Darmfunktion im Rahmen einer GDT zeigen [12, 125, 136]. Zudem gibt es erste Daten, dass eine verminderte Flüssigkeitsgabe durch GDT im Bereich der Leberchirurgie Vorteile für die Patienten hat, denn besonders in diesem Bereich sollte aufgrund der Komplexität der einzelnen Phasen immer ein individuelles Vorgehen erfolgen [33, 142]. Auf Basis dieser Positivergebnisse sprechen sich französische und britische Leitlinien insbesondere bei Risikopatienten aktuell für die Verwendung einer GDT aus [96, 132].

##### Merke

Vorgehen für die intraoperative Phase

- Ziel ist die Euvolämie („Nullbilanz“),
- balancierte Kristalloide als Basistherapie mit Einsatz von niedrigdosierten Katecholaminen zur Kompensation von Volumenverschiebungen,
- an Patient und Operation angepasstes Überwachungsverfahren.

### Postoperative Phase

**Figure 4:**
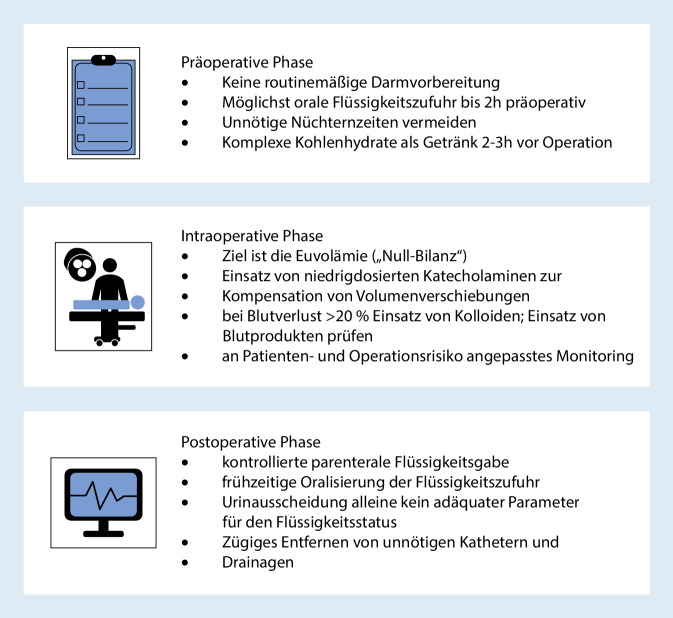

Die postoperative Phase nimmt im Rahmen des perioperativen Flüssigkeitsmanagements eine entscheidende Rolle ein. Viele der zitierten Studien, die positive Ergebnisse für ein restriktives Management zeigen konnten, haben die Restriktion auch in der ersten postoperativen Phase weitergeführt (Tab. 2). Letzteres macht deutlich, dass die Vorteile einer durchdachten Flüssigkeitsgabe während der Operation möglicherweise nur in einem konsequenten perioperativen Gesamtkonzept sichtbar werden bzw. durch inadäquate postoperative Flüssigkeitszufuhr negativ beeinträchtigt werden könnten. Für diese Phase scheint deshalb eine dezidierte Übergabe durch den betreuenden Anästhesisten mit Blick auf den postoperativen Volumenstatus sinnvoll. Eventuelle Flüssigkeitsüberschüsse/-mängel sollten evaluiert und in die Therapieplanung mitaufgenommen werden. Zur Vermeidung einer unkontrollierten Zufuhr sollte der betreuende Arzt zu Beginn der postoperativen Überwachung die anzustrebende Bilanzierung festlegen und diese in regelmäßigen Abständen reevaluieren. Dabei kann im postoperativen Setting auf Verfahren zurückgegriffen werden, deren Durchführung intraoperativ evtl. nicht möglich war, z. B. eine transthorakale Echokardiographie. Sobald der Patient wach genug ist und keine chirurgischen Kontraindikationen bestehen, sollten zudem frühzeitig mit der Oralisierung begonnen und die i.v.-Flüssigkeitszufuhr angepasst werden [37, 81, 110]. Abb. 4 fasst die Eckpfeiler des perioperativen Flüssigkeitsmanagements zusammen.

#### Merke

Vorgehen für die postoperative Phase

- Reevaluation der Flüssigkeitsbilanz
- bei stabilen Patienten und ausgeglichenem Flüssigkeitsstatus schnelle Oralisierung anstreben

## Fazit für die Praxis

Bei viszeralchirurgischen Eingriffen scheinen das Ziel der Euvolämie und ein Vermeiden perioperativer Flüssigkeitseinlagerung mit dem besten Behandlungsergebnis einherzugehen. Zur Therapiesteuerung reicht bei kleinen und mittelgroßen Eingriffen (z. B. einer Hemikolektomie) ein Basis-Monitoring, inklusive Messung der Diurese, aus, sofern der Allgemeinzustand des Patienten dies zulässt. Bei Eingriffen an Pankreas, Magen oder in der hepatobiliären Chirurgie wird dieses um eine invasive, kontinuierliche Messung von Blutdruck und zentralem Venendruck (ZVD) sowie um Blutgasanalysen erweitert. An dieser Stelle sollten je nach Verfügbarkeit auch Verfahren zur Messung der Pulsdruckvariabilität zur Einschätzung des Volumenbedarfs herangezogen werden, da diese keines invasiveren Vorgehens bedürfen. In berechtigten Einzelfällen kommt zusätzliches Monitoring (z. B. PICCO®, TEE) zum Einsatz, ergänzt durch Notfalllabor- bzw. *Point-of-care*-Gerinnungsanalysen (z. B. ROTEM®[TEM Innovations GmbH, München, Deutschland]- und Multiplate® [Roche Deutschland Holding GmbH, Grenzach-Wyhlen, Deutschland]).

Die Basistherapie zum Ausgleich von Flüssigkeitsverlusten fußt auf balancierten Kristalloiden. Narkosebedingte Auswirkungen auf die Hämodynamik (z. B. Sympathikolyse bei Periduralanästhesie) sollten frühzeitig durch ergänzende Gabe von Vasopressoren (i. d. R. Noradrenalin 0,05–0,1 µg/kgKG und min) kompensiert werden. Reicht dies nicht aus, wird nach Reevaluation von Blutverlust, bereits infundierter Kristalloidmenge, Katecholaminbedarf und Verlauf der Blutgasanalysen ggf. ein zügiger Flüssigkeitsbolus von ca. 200–300 ml verabreicht. Erhärtet sich der Verdacht einer persistierenden Hypovolämie, kann in einer nächsten Stufe auf Kolloide (z. B. Humanalbumin 20 %) zurückgegriffen werden. Das in der zitierten Übersichtsarbeit von Rehm et al. beschriebene Dreistufenkonzept zur bedarfsorientierten Volumentherapie kann hier eine wertvolle Orientierung bieten. Unter individueller Indikationsstellung wird die Therapie durch Gabe von Blutprodukten erweitert. Bei ansonsten gesunden Patienten ist es so möglich, beispielsweise eine komplikationslose Whipple-Operation mit einer Kristalloidgabe von weniger als 2 l durchzuführen.

Entscheidend ist, dass ein adäquates Flüssigkeitsmanagement bereits präoperativ beginnt und auch in der postoperativen Phase fortgeführt werden sollte. Zur Vermeidung einer unkontrollierten Flüssigkeitsgabe kommt der Kommunikation und der gemeinsamen Zielsetzung aller an der perioperativen Versorgung Beteiligten eine entscheidende Bedeutung zu.

## Abkürzungen

ADH: Antidiuretisches Hormon
ASA: American Society of Anesthesiologists
C: „Conventional“
EMA: European Medicines Agency
ESA: European Society of Anaesthesiology
GDT: Goal-directed therapy
GOR: Grade of recommendation
H: „Hypertonic saline“
HES: Hydroxyethylstärke
L: Liberal
NaCl: Natriumchlorid
R: Restriktiv
RAAS: Renin-Angiotensin-Aldosteron-System
RL: Ringer-Laktat-Lösung
S: Standard
SVV: Schlagvolumenvariabilität
TEE: Transösophageale Echokardiographie
ZVD: Zentraler Venendruck
